# PD-1/PD-L1 blockade as part of combination strategies toward functional cure of chronic hepatitis B

**DOI:** 10.3389/fimmu.2026.1831739

**Published:** 2026-07-09

**Authors:** Mei Li, Dandan Feng, Juanjuan Shi, Shuangsuo Dang, Xiaoli Jia, Wenjun Wang

**Affiliations:** Department of Infectious Diseases, The Second Affiliated Hospital of Xi’an Jiaotong University, Xi’an, China

**Keywords:** chronic hepatitis B, functional cure, HBsAg, HBV, immune checkpoint inhibition, nucleos(t)ide analogue, PD-1, PD-L1

## Abstract

Chronic hepatitis B (CHB) affects 260 million people worldwide. Despite advances in antiviral therapies, functional cure, characterized by sustained loss of hepatitis B surface antigen (HBsAg) and durable viral control after treatment cessation, remains infrequent. The PD-1/PD-L1 immune checkpoint pathway plays an important role in the exhaustion of hepatitis B virus (HBV)-specific T cells, thereby limiting the immune system’s ability to clear HBV. Blocking the PD-1/PD-L1 pathway has emerged as a promising strategy to overcome this immune exhaustion to some extent and reinvigorate antiviral T-cell responses in CHB patients. Early clinical trials, particularly among patients who have achieved viral suppression with nucleos(t)ide analogues, have demonstrated that PD-1/PD-L1 inhibitors can significantly reduce HBsAg levels. Notably, a subset of patients has achieved functional cure, particularly among those with low baseline HBsAg levels. These effects have been further enhanced in combination with pegylated interferon, resulting in a functional cure rate of 30%. While challenges remain, such as optimizing dosing regimens, selecting patients, identifying response predictors and managing immune-related adverse events, these findings emphasize the potential of combination regimens involving PD-1/PD-L1 blockade as a means of achieving a functional cure in CHB patients. Future large-scale randomized controlled trials are essential to fully assess the approach’s efficacy and safety, and to refine its clinical application.

## 1 Introduction

Chronic hepatitis B (CHB) continues to affect approximately 260 million people worldwide despite effective prophylactic vaccination and remains a leading cause of cirrhosis and hepatocellular carcinoma (1). Current standard therapies include nucleos(t)ide analogues (NAs) and pegylated interferon (Peg-IFN) (2–4). NAs reliably suppress hepatitis B virus (HBV) replication and reduce liver-related complications (5, 6), but most patients require long-term, often indefinitely, treatment to maintain viral suppression (2–4). Viral persistence is sustained by covalently closed circular DNA (cccDNA) and integrated HBV DNA, both of which can continue to support viral antigen production despite the effective suppression of serum HBV DNA (7–9). Peg-IFN exerts antiviral and immunomodulatory effects by inducing interferon-stimulated genes and enhancing innate and adaptive immune responses (10). It may promote cccDNA silencing or reduction in certain cases, but it is unable to eliminate or specifically silence integrated HBV DNA (11, 12). Therefore, although Peg-IFN offers a finite treatment course and can accelerate hepatitis B surface antigen (HBsAg) decline or clearance in selected patients, its overall efficacy remains limited and its tolerability constrains its widespread use (11–16).

Functional cure and complete sterilizing cure represent two distinct therapeutic endpoints in CHB. Functional cure is generally defined as sustained HBsAg loss, with or without seroconversion of antibody to hepatitis B surface antigen (anti-HBs), accompanied by durable suppression of HBV replication after treatment cessation (17, 18). This endpoint is clinically meaningful as HBsAg loss is associated with a reduced risk of cirrhosis and hepatocellular carcinoma (19–21), as well as the potential for finite therapy in certain patients. By contrast, complete sterilizing cure would require the elimination of all cccDNA and integrated HBV DNA from infected hepatocytes (22). Such complete eradication remains elusive with the therapies currently available. Therefore, the realistic aim of emerging cure-directed strategies is not an immediate sterilizing cure, but rather the establishment of durable immune-mediated control of residual viral reservoirs following the effective suppression of viral replication and reduction of antigen burden (22).

The difficulty of curing CHB is largely due to profound and persistent immune dysfunction. Prolonged exposure to viral antigens, including HBsAg and subviral particles, drives HBV-specific T cells toward an exhausted state characterized by reduced proliferative capacity, diminished effector function, and increased expression of inhibitory receptors (23–26). Among these pathways, programmed cell death protein 1 (PD-1) and its ligand PD-L1 represent a central checkpoint axis that restricts antiviral immunity and limits the clearance of infected hepatocytes (24, 27). This provides a strong mechanistic rationale for exploring PD-1/PD-L1 blockade as a strategy to restore antiviral immunity.

Importantly, PD-1/PD-L1 blockade should not be viewed as a replacement for optimized standard-of-care therapy or as a standalone curative intervention. PD-1/PD-L1 inhibitors pose a moderate risk of HBV reactivation in HBsAg-positive individuals who are not receiving antiviral prophylaxis with NAs (28, 29). Furthermore, all published clinical studies of PD-1/PD-L1 blockade in CHB have been conducted alongside NA therapy or in conjunction with other immune interventions, such as therapeutic HBV vaccines or Peg-IFN (30–36). Therefore, checkpoint blockade is most appropriately considered as an investigational, immune-amplifying adjunct within rational combination regimens that provide viral suppression, antigen reduction, immune priming, and checkpoint modulation.

In this review, we summarize the mechanistic rationale and emerging clinical evidence for PD-1/PD-L1 blockade in CHB, discuss predictive biomarkers and safety considerations, and highlight key challenges that remain to be resolved for future clinical development.

## 2 Mechanistic rationale: breaking immune tolerance

**Figure 1:**
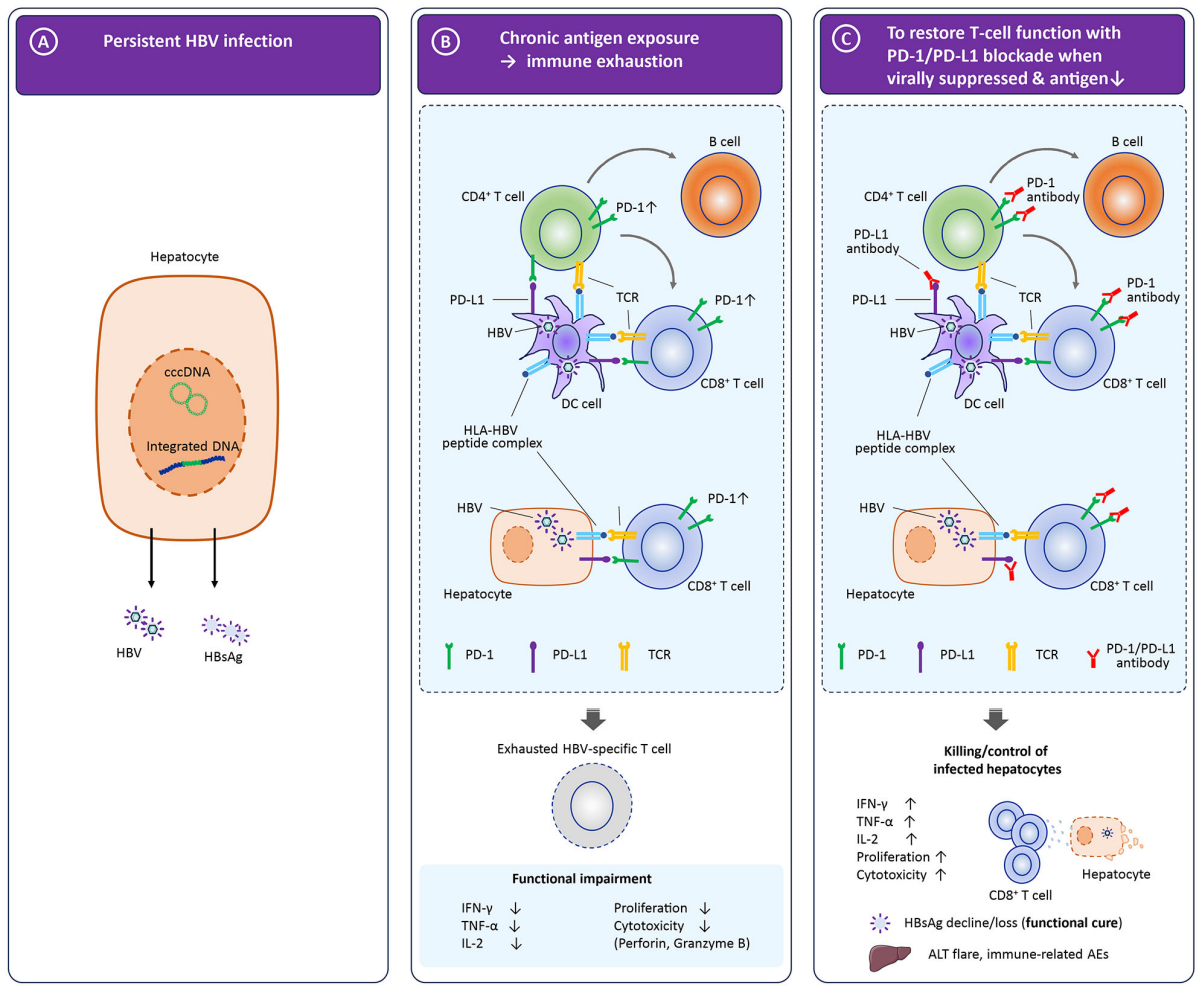
Mechanistic rationale of PD-1/PD-L1 blockade for the functional cure of CHB. **(A)** Persistent HBV infection is maintained by cccDNA and integrated HBV DNA, both of which contribute to ongoing antigen production. **(B)** During chronic antigen exposure, HBV-infected hepatocytes process intracellular viral proteins and present HBV-derived peptides through HLA class I molecules. These HLA-HBV peptide complexes are recognized by T-cell receptors on HBV-specific CD8+ T cells. Antigen presentation by dendritic cells also coordinates CD4+ and CD8+ T-cell responses and supports B-cell immunity. However, persistent antigen stimulation within the tolerogenic liver microenvironment promotes PD-1 upregulation and PD-L1-mediated inhibitory signaling, resulting in HBV-specific T-cell exhaustion. This state is characterized by reduced IFN-γ, TNF-α, and IL-2 production, impaired proliferation, and diminished cytotoxicity. **(C)** When viral replication is suppressed and the antigen burden is reduced, PD-1/PD-L1 blockade may partially restore CD8+ and CD4+ T-cell function, enhance cytokine production, proliferation, and cytotoxicity, and support B-cell antibody responses. These immune effects may improve the killing or control of infected hepatocytes and promote HBsAg decline or loss, thereby contributing to functional cure. Nevertheless, treatment may also be associated with ALT flares and immune-related adverse events. ALT, alanine aminotransferase; CHB, chronic hepatitis B; cccDNA, covalently closed circular DNA; HBsAg, hepatitis B surface antigen; HBV, hepatitis B virus; HLA, human leukocyte antigen; IL-2, interleukin-2; IFN-γ, interferon-γ; PD-1, programmed cell death protein 1; PD-L1, programmed death-ligand 1; TCR, T-cell receptor; TNF-α, tumor necrosis factor-α.

CHB is defined not only by virologic persistence but also by a distinct immune landscape shaped by prolonged exposure to viral antigens and the inherently tolerogenic environment of the liver (37). In acute-resolving HBV infection, broad and multifunctional CD8+ and CD4+ T-cell responses cooperate with innate immune signals and neutralizing antibodies to achieve viral clearance (24, 26, 38). By contrast, chronic infection is characterized by quantitative and qualitative defects across multiple immune compartments, including dysfunctional HBV-specific T cells, impaired antigen presentation, altered natural killer cell function, and the expansion of regulatory or suppressive pathways (24, 26, 38). Within this network, the PD-1/PD-L1 axis is one of the most extensively characterized inhibitory pathways. PD-1 is highly expressed on exhausted HBV-specific T cells, whereas PD-L1 is expressed by hepatocytes, liver sinusoidal endothelial cells, dendritic cells, Kupffer cells, and other antigen-presenting cells (24, 39). Engagement of PD-1/PD-L1 limits T-cell receptor (TCR) signaling, suppresses effector cytokine production, and stabilizes transcriptional programs of exhaustion (40, 41). Therefore, PD-1/PD-L1 blockade aims to restore antiviral effector function and help the host immune system regain control of HBV-infected hepatocytes (**Figure 1**).

### 2.1 Antigen recognition, persistent stimulation, and T-cell exhaustion

HBV-specific CD8+ T cells recognize infected hepatocytes via human leukocyte antigen (HLA) class I-restricted antigen presentation, rather than by binding to free viral antigens directly. Intracellular HBV-derived proteins are processed by infected hepatocytes and presented as short peptides via HLA class I molecules. These HLA-HBV peptide complexes are recognized by the TCR on HBV-specific CD8+ T cells, enabling the identification and destruction of infected hepatocytes (42, 43). However, in chronic infection, continuous antigen presentation within the tolerogenic hepatic microenvironment drives sustained TCR stimulation and progressive immune dysfunction (44, 45).

HBV-specific CD8+ T cells in CHB are phenotypically exhausted (46). Exhaustion is not a binary state, but rather a spectrum in which cells may retain partial effector capacity while being constrained by inhibitory receptors, metabolic disturbances, and rigid epigenetic programs (46, 47). Persistent exposure to HBV antigens, including HBsAg and subviral particles, promotes the upregulation of inhibitory receptors such as PD-1, while PD-L1 expression on hepatic and immune cells reinforces inhibitory signaling (39, 47). This process reduces the production of interferon-γ (IFN-γ), tumor necrosis factor-α (TNF-α), and interleukin-2 (IL-2), impairs proliferation, diminishes cytotoxicity, and limits durable immune control (37, 39–41). Although NAs therapy effectively suppresses HBV DNA replication, the reduction of circulating HBsAg and subviral particles is often slow and incomplete (3, 22). Therefore, PD-1/PD-L1 blockade is most likely to be effective when the antigen burden is already low or is actively being reduced by agents such as NAs, Peg-IFN, and emerging therapies such as small interfering RNA (siRNA) and antisense oligonucleotide (ASO) (22, 48).

### 2.2 Molecular basis of PD-1/PD-L1 blockade

At the molecular level, PD-1/PD-L1 engagement inhibits proximal TCR signaling in HBV-specific CD8+ and CD4+ T cells. This results in reduced phosphorylation of CD3zeta and ZAP-70, as well as attenuated activation of the downstream PI3K-AKT and Ras-MEK-ERK pathways (49–51). These changes suppress the production of effector cytokines and the release of cytotoxic granules. Blocking PD-1/PD-L1 can partially reverse these inhibitory signals, thereby enhancing T-cell proliferation, cytokine secretion, and cytotoxicity (41). However, the extent to which T cells are reinvigorated depends on their differentiation state. Those with reversible or intermediate exhaustion phenotypes are more likely to respond than terminally exhausted populations with fixed epigenetic programs (22, 41, 46).

### 2.3 Role of CD4+ T cells and humoral immunity in functional cure

Functional cure is generally defined as sustained HBsAg loss with or without anti-HBs seroconversion. The appearance of anti-HBs represents a more complete immunological endpoint because it reflects the restoration of HBV-specific humoral immunity. In this context, CD4+ T cells play a central role in durable immune control. As well as supporting the differentiation, survival, and memory formation of CD8+ T-cells, CD4+ T cells promote B-cell responses through germinal center formation, CD40L-CD40 interactions, isotype switching, affinity maturation, and cytokine production, particularly IL-21 (38, 52, 53). These processes are essential for generating high-quality anti-HBs antibodies and durable immune memory (52).

Recent clinical immunology data support this concept. Hoogeveen et al. demonstrated that HBV-specific CD4+ T-cell responses differentiate functional cure from chronic HBsAg-positive infection. Patients with functional cure exhibited higher frequencies of functional HBV-specific CD4+ memory T-cell responses than those with persistent chronic infection, whereas differences in CD8+ T-cell responses were less prominent (53). These findings suggest that CD4+ T-cell immunity is a key correlate of durable HBV control and should be incorporated into the mechanistic framework of immunotherapeutic cure strategies. PD-1/PD-L1 blockade may therefore contribute to functional cure not only by reinvigorating exhausted CD8+ T cells, but also by restoring CD4+ T-cell helper activity. This would support therapeutic vaccine-induced T-cell responses, B-cell maturation, anti-HBs development, and long-term immune memory (38, 52, 53).

### 2.4 Combination strategies to overcome immune bottlenecks

No currently available therapy is sufficient to cure most patients with CHB, including PD-1/PD-L1 blockade. Therefore, rational combination strategies aim to address complementary barriers, such as viral replication, antigen excess, insufficient immune priming and checkpoint-mediated exhaustion (22). In this context, NAs suppress HBV DNA replication to provide an antiviral backbone, while agents that lower antigens, such as siRNA and ASO, reduce HBsAg burden. Peg-IFN provides direct antiviral and immunomodulatory activity, while therapeutic vaccines prime or enhance HBV-specific T-cell responses. Finally, PD-1/PD-L1 blockade releases inhibitory signaling to amplify pre-existing or vaccine-induced antiviral immunity.

Preclinical and clinical evidence supports this combination approach. Meng et al. demonstrated in animal models that liver-targeted anti-PD-L1-IFN-α fusion proteins can bridge innate and adaptive immunity, helping to break HBV immune tolerance (54). Clinically, Tak et al. reported that VTP-300, a heterologous ChAdOx1-HBV/MVA-HBV therapeutic vaccine, induced CD4+ and CD8+ T-cell responses and reduced HBsAg in a subset of patients. The addition of low-dose nivolumab produced more pronounced HBsAg declines and HBsAg loss in selected patients (36). Gane et al. also demonstrated that low-dose nivolumab, with or without GS-4774 therapeutic vaccination, was well tolerated and associated with HBsAg decline in virally suppressed patients (30). Together, these findings suggest that therapeutic vaccines and checkpoint blockade may act cooperatively. Therapeutic vaccines prime HBV-specific T-cell responses, whereas PD-1/PD-L1 blockade reinvigorates exhausted vaccine-induced or pre-existing T cells. This provides a mechanistic foundation for incorporating checkpoint inhibitors into combination regimens rather than using them as a standalone therapy.

## 3 Clinical efficacy of PD-1/PD-L1 blockade in CHB

At present, seven clinical studies provide the primary evidence for PD-1/PD-L1 blockade in CHB treatment (30–36). A defining feature of these studies is that all patients were virally suppressed on long-term NAs therapy prior to PD-1/PD-L1 blockade therapy, with NAs treatment maintained throughout study treatment. This design minimizes HBV reactivation risk while allowing immune restoration to result in HBsAg decline, as PD-1/PD-L1 inhibitors are classified as a moderate-risk exposure for HBV reactivation in HBsAg-positive individuals without adequate antiviral prophylaxis (28, 29).

Although these studies vary substantially in terms of design, dosing intensity, treatment duration, and concomitant therapies, they collectively provide meaningful signals toward functional cure. HBsAg loss rates remain heterogeneous and modest in unselected populations, but responses are enriched in patients with low baseline HBsAg levels, particularly those below 100 IU/mL. **Table 1** summarizes the detailed dose, duration, background NAs therapy, combination partners, HBsAg decline, and functional cure rate.

**Table 1:** Characteristics of seven clinical studies evaluating PD-1/PD-L1 blockade for the treatment of CHB.

Study and clinical trial ID	Design	CHB patients	Intervention and sample size	PD-1/PD-L1 course	Age, y	Sex (M/F)	NA	Baseline DNA, IU/mL	Baseline HBsAg (log_10_ IU/mL)	HBsAg decline	Functional cure rate
Gane 2019 (30) ACTRN12615001133527	Phase 1b, non-RCT	Virally suppressed with NAs	A: NIV (0.1 mg/kg), n=2 B: NIV (0.3 mg/kg), n=12 Arm C: NIV (0.3 mg/kg) + GS-4774, n=10	Single dose	A: 50 (44–55) B: 51 (41–63) C: 54 (38–64)^#^	A: 1/1 B: 9/3 C: 8/2	All NA	<20	A: 3.1 (3.0-3.2) B: 2.7 (1.0-4.0) C: 2.6 (1.9-3.6)^#^	A: 0.01 (-0.38, 0.40) B: -0.30 (-0.46, -0.14) C: -0.16 (-0.33, 0.01), at week 12	A: 0/2 B: 1/12 C: 0/10 4.2% overall
Tak 2024 (36) NCT04778904	Phase 1b/2a, RCT	Virally suppressed with NAs	A: MVA-HBV, MVA-HBV, n=9 B: ChAdOx1-HBV, MVA-HBV, n=18 C: ChAdOx1-HBV, MVA-HBV + NIV (0.3 mg/kg), n=18 D: ChAdOx1-HBV + NIV (0.3 mg/kg), MVA-HBV + NIV (0.3 mg/kg), n=9	C: 1 dose D: 2 doses	A: 50.8±8.8 B: 53.3±6.9 C: 49.8±8.5 D: 49.9±9.7^§^	A: 8/1 B: 15/3 C: 11/7 D: 7/2	All NA	<40	A: 2.4±0.6 B: 2.4±0.9 C: 2.5±0.7 D: 2.7±0.5^§^	A: -0.1±0.1 B: -0.2±0.4 C: -0.8±1.1 (p<0.001 from baseline) D: -0.2±0.2, at month 3	A: 0/9 B: 0/18 C: 2/18 D: 0/9 7.4% overall
He 2025 (31) NCT05769816	Phase 2, non-RCT	Virally suppressed with NAs	A: none, n=62 B: sintilimab (100 mg, every 3 weeks), n=59	24 weeks (8 cycles)	A: 48 (24–67) B: 46 (31–60)^*^	A: 46/16 B: 49/10	All NA	<20	A: 2.4 (2.0-2.7) B: 2.4 (2.0-2.7) ^#^	A: -0.034 B: -0.720, at week 24, p<0.001 between groups	A: 0/62 B: 3/49 6.1% overall
Qian 2025 (34) NCT04465890	Phase 2b, RCT	Virally suppressed with NAs	A: placebo, n=30 B: ASC22 (1.0 mg/kg, every 2 weeks), n=60 C: ASC22 (2.5 mg/kg, every 2 weeks), n=59	24 weeks (12 cycles)			All NA	<20		A: -0.003 from baseline B: -0.309 (p<0.001 from baseline) C: -0.231 (p=0.007 from baseline) at week 24	A: 0/30 B: 3/60 C: 0/59 2.5% overall
Mon 2025 (33)	Real-world	Cancer patients with CHB, most with NAs	A: pan-cancer, PD-1/PD-L1 inhibitor, n=118 B: HCC, PD-1/PD-L1 inhibitor, n=44 C: HCC, TKI, n=85	A: 11 cycles B: 11 cycles	A: 57 (51–64) B: 60 (54–67) C: 62 (52–72)^#^	A: 90/28 B: 42/2 C: 73/12	A:108 NA B: 44 NA C: 78 NA	A: 65 (<10, 3850) B: <10 (<10, 7040) C: 19 (<20, 2290)^#^	A: 2.6 (1.8-3.2) B: 1.9 (1.6-2.7) C: 2.7 (2.0-3.2)^#^	A: 6.6% B: 22.7% C: 2%, HBsAg loss/decline >1 log at month 24	A: 4.3% B: 16.9% C: 0% at month 24
Qin 2025 (35) NCT04233840	Phase 1	HCC after resection	Ropeg every 2 weeks (6 doses), followed by NIV (0.3-0.75 mg/kg) every 2 weeks, n=15	6 weeks (3 cycles)	61.8±10.8^§^	11/4	All NA	Undetectable	3.6±3.4^§^	No mention	3/15 20.0% overall
Li 2026 (32) ChiCTR2400091948	Non-RCT	Virally suppressed with NAs, prior Peg-IFN failure	A: Peg-IFN + sintilimab (1 mg/kg, every 12 weeks), n=33 B: Peg-IFN, n=31	24 weeks (2 cycles)	A: 42.8±10.1 B: 45.8±10.5^§^	A: 21/12 B: 21/10	All NA	<20	A: 0.9 (0.3-1.9) B: 0.4 (0.1-1.9)^#^	A: 0.03 at w24(p=0.007 from baseline) B: 0.4 at week24 (p>0.05 from baseline)	A: 10/33 B: 2/31 30.3% overall

CHB, chronic hepatitis B; HBsAg, hepatitis B virus surface antigen; HBV, hepatitis B virus; HCC, hepatocellular carcinoma; NAs, nucleos(t)ide analogues; Peg-IFN, pegylated interferon; RCT, randomized controlled trial; TKI, tyrosine kinase inhibitor. ^*^ mean (range); ^#^ median (range); ^§^ mean±standard deviation.

### 3.1 Single-dose pilot studies: proof of concept

In a phase Ib pilot study, Gane et al. demonstrated that even a single low dose of nivolumab (0.1–0.3 mg/kg) could trigger a decline in HBsAg in virally suppressed patients (30). This clinical event was preceded by a significant alanine aminotransferase (ALT) flare and a concomitant expansion of peripheral HBV-specific T cells. Overall, one of the 24 (4.2%) patients treated with nivolumab achieved HBsAg loss by week 16.

Building on this concept, Tak et al.’s phase Ib/IIa study evaluated a single dose of nivolumab (0.3 mg/kg) alongside the VTP-300 therapeutic vaccine (36). This combination therapy significantly enhanced HBsAg reduction (mean decline ~0.8 log10 IU/mL) compared to the vaccine alone, with the most pronounced responses observed in patients with baseline HBsAg <100 IU/mL. Notably, two of the 27 (7.4%) nivolumab-treated patients achieved HBsAg loss at months 3 and 9, respectively. Taken together, these pilot studies provide pivotal proof of concept that even transient checkpoint interference can disrupt immune tolerance and reactivate a dormant antiviral response in certain patients.

### 3.2 Repeated dosing: sustained immune pressure

Advancing beyond single-dose pilot studies, repeated administration strategies have been investigated to maintain longitudinal immune pressure and overcome the robust tolerance characteristic of the liver microenvironment.

Cancer patients who are HBsAg-positive and receiving standard-intensity PD-1/PD-L1 blockade for malignancy offer a unique clinical perspective on the effects of sustained checkpoint inhibition. A real-world study has shown that a functional cure can be achieved even within the complex immune landscape of oncology patients (33). Among those with baseline HBsAg <100 IU/mL, the cumulative incidence of HBsAg loss was 13.0% at 12 months and 38.4% at 24 months. Although the immune status of oncology patients is characterized by tumor-induced exhaustion and concurrent systemic therapies, which differ significantly from non-oncology CHB populations, these findings provide critical evidence that prolonged checkpoint blockade can drive HBsAg seroclearance, particularly when the viral antigen burden is low.

In a prospective phase II study, virally suppressed CHB patients received 24 weeks of half-dose sintilimab (anti-PD-1) every three weeks (31). This regimen resulted in a significantly greater mean HBsAg decline compared to NA monotherapy (-0.720 vs -0.034 log_10_ IU/mL; *P* < 0.001). The finding that HBsAg clearance (6.1%) was often preceded by transient ALT flare indicates a certain level of hepatic inflammation may be essential for successful immune-mediated viral clearance. The randomized phase 2b trial of ASC22 (a subcutaneous PD-L1 antibody) further refined this approach (34). The 1.0 mg/kg biweekly dose was more effective than the 2.5 mg/kg dose, achieving HBsAg loss in 30% of patients with baseline HBsAg <100 IU/mL. This “less-is-more” phenomenon suggests that, for CHB, there may be an optimal therapeutic window in which immune restoration can be achieved without triggering excessive counter-regulatory mechanisms.

### 3.3 Combination with therapeutic vaccine

Unlike prophylactic vaccines, which aim to prevent HBV infection, therapeutic vaccines are designed to restore or enhance HBV-specific immune responses in patients with an established chronic infection (55, 56). In cure-directed combination regimens, therapeutic vaccines may prime HBV-specific T-cell responses, while PD-1/PD-L1 blockade may help reinvigorate exhausted vaccine-induced or pre-existing T cells (54).

Recent clinical evidence suggests that PD-1/PD-L1 blockade could have a synergistic effect with therapeutic HBV vaccines in cases of chronic infection (30, 36). While therapeutic vaccination can prime HBV-specific T cells, vaccine efficacy alone is limited by T-cell exhaustion and the immunosuppressive intrahepatic microenvironment. PD-1/PD-L1 blockade may amplify these vaccine-induced T-cell responses by enhancing proliferation, cytokine production, and cytotoxic activity. The VTP-300 study found that adding low-dose nivolumab resulted in a more pronounced HBsAg decline than the vaccine alone (36). The pilot study of nivolumab with or without GS-4774 provided early proof of concept for combining checkpoint blockade with therapeutic vaccination in patients with suppressed virus (30). These findings provide mechanistic and translational justification for combining therapeutic vaccines with checkpoint inhibition in selected patients with suppressed viral replication and reduced antigen burden.

### 3.4 Combination with Peg-IFN

Integrating Peg-IFNα with PD-1/PD-L1 blockade appears to be the most potent immunotherapeutic strategy to date. Peg-IFNα exerts a bimodal antiviral effect. It induces interferon-stimulated genes (ISGs), which directly promote the degradation of HBV pregenomic RNA and core particles. At the same time, it reshapes the hepatic immune landscape by enhancing major histocompatibility complex expression and natural killer cell activity (57–59). However, many patients remain refractory to IFN therapy, experiencing a plateau in HBsAg decline without achieving seroclearance (15, 60).

Our team’s research may explain this therapeutic resistance: IFN-induced adaptive immune resistance (61). We found that patients undergoing Peg-IFNα therapy had significantly higher proportions of PD-1+ lymphocytes and CD8+PD-1+ T cells than both healthy controls and non-IFN-treated patients (*P* < 0.001). Specifically, after 48 weeks of Peg-IFNα therapy, the proportion of PD-1+ lymphocytes was almost double that of the non-IFN group (24.3% vs 12.7%, *P* < 0.01), with a similar increase in CD8+PD-1+ T cells (29.3% vs 17.7%, *P* < 0.05). Importantly, higher PD-1 expression within the IFN-treated group was associated with poorer HBsAg decline (61). This suggests that, while IFN activates the immune system, it simultaneously triggers the PD-1/PD-L1 pathway as a compensatory brake, thereby limiting its own efficacy. Blocking this axis may therefore be a rational rescue strategy to reinvigorate the immune response in IFN-refractory patients.

Two studies have explored the sequential or synchronized combination of IFN and PD-1 blockade to maximize functional cure rates (32, 35). One study focused on preventing cancer recurrence following resection of HBV-related hepatocellular carcinoma. Fifteen patients received six doses of ropeginterferon alfa-2b, followed by three escalating doses of nivolumab (0.3 to 0.75 mg/kg) (35). ALT flare occurred in five patients, correlating with HBsAg decline, and three patients achieved functional cure. In this sequential strategy, Peg-IFN primed the immune environment, while PD-1 blockade acted as the “final push” to achieve seroclearance. The most compelling evidence for this synergy comes from a trial in CHB patients who had failed to achieve HBsAg clearance after 48 weeks of Peg-IFNα therapy (32). Patients were assigned to either continue IFN monotherapy (n = 31) or receive a synchronized combination of sintilimab (1 mg/kg every 12 weeks) plus Peg-IFNα (n = 33). At week 24, the combination group achieved an HBsAg clearance rate of 30.3%, which was significantly higher than the 9.7% rate observed in the Peg-IFNα-only group (P = 0.047). Furthermore, the median HBsAg decline in the combination group was almost 14 times greater than that of the monotherapy group (-0.916 vs -0.067 log_10_ IU/mL; *P* = 0.013). These results confirm that adding a PD-1 inhibitor can successfully transform IFN-refractory cases into functional cures.

### 3.5 Clinical positioning of PD-1/PD-L1 blockade in CHB treatment

**Figure 2:**
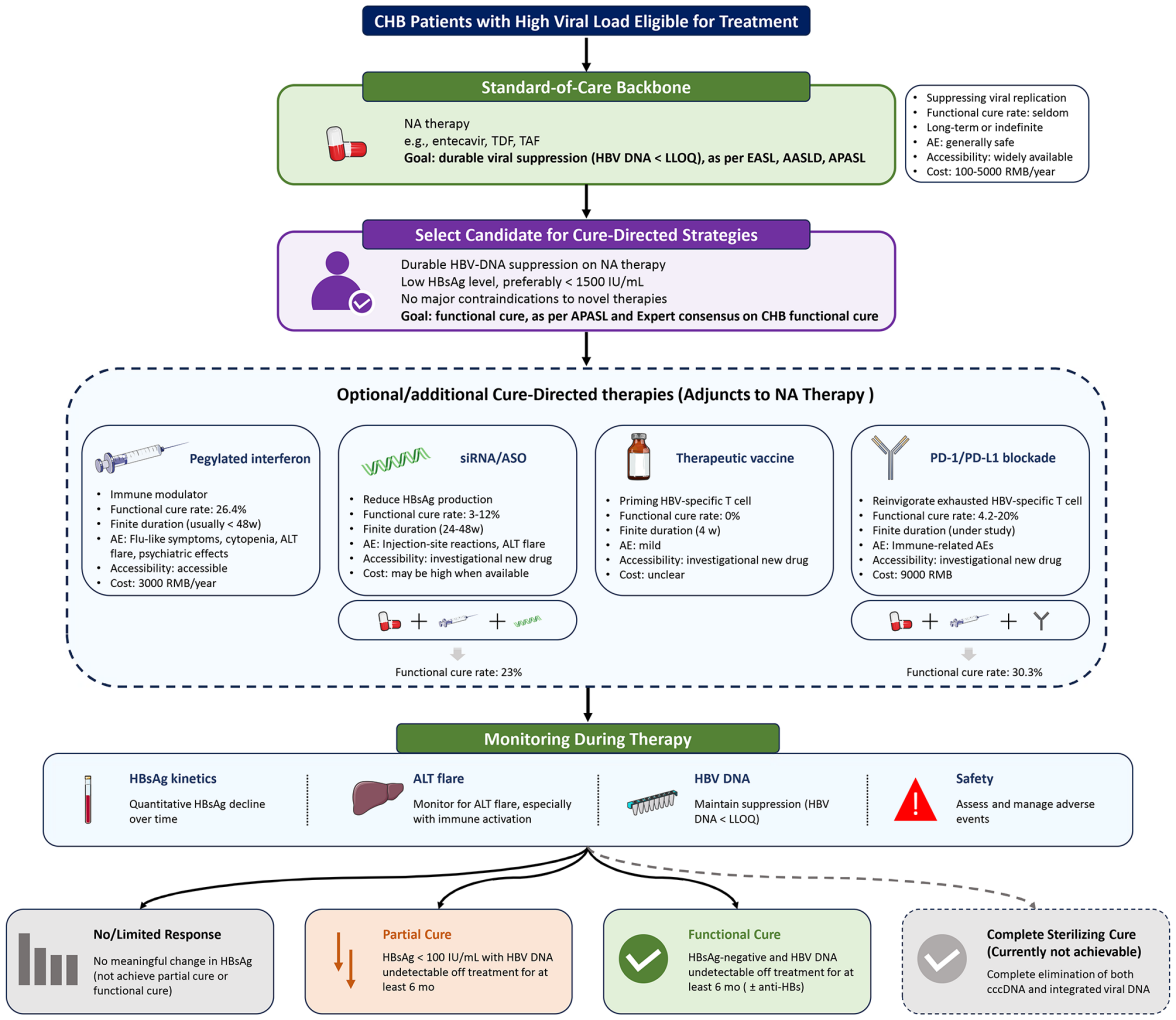
The clinical positioning of PD-1/PD-L1 blockade within cure-directed treatment strategies for CHB. NAs therapy forms the standard-of-care backbone for durable viral suppression. Peg-IFN, siRNA/ASO-based therapies, therapeutic vaccines, and PD-1/PD-L1 blockade are optional or investigational adjuncts which target immune activation, antigen reduction, immune priming, and checkpoint modulation. This figure summarizes patient selection, treatment rationale, expected outcomes, adverse events, and monitoring. Functional cure is defined as HBsAg negativity and undetectable HBV DNA off treatment for at least 6 months, with or without anti-HBs seroconversion. Complete sterilizing cure, which requires the elimination of both cccDNA and integrated viral DNA, remains unattainable currently. AE, adverse event; ALT, alanine aminotransferase; ASO, antisense oligonucleotide; CHB, chronic hepatitis B; cccDNA, covalently closed circular DNA; HBsAg, hepatitis B surface antigen; HBV, hepatitis B virus; LLOQ, lower limit of quantification; NA, nucleos(t)ide analogue; PD-1, programmed cell death protein 1; PD-L1, programmed death-ligand 1; Peg-IFN, pegylated interferon; siRNA, small interfering RNA.

Based on the available clinical evidence, PD-1/PD-L1 blockade appears to be most effective when used as an adjunct in combination with other treatments aimed at achieving a cure, rather than as a standalone therapy or a replacement for the current standard of care (**Figure 2**). NAs remain the therapeutic backbone as they provide durable viral suppression and reduce the risk of HBV reactivation during immune modulation (2–4, 22, 28). Within this framework, PD-1/PD-L1 blockade could be considered for carefully selected patients who have achieved viral suppression and a low antigen burden. This is particularly pertinent for individuals with low HBsAg levels or incomplete HBsAg clearance following Peg-IFN or other antigen-lowering interventions. As shown in **Figure 2**, its potential role is to provide immune amplification following viral suppression, antigen reduction, or immune priming, thereby increasing the likelihood of HBsAg loss. However, as current evidence is limited to early-phase or selected clinical settings, this strategy should still be regarded as investigational and requires validation in large randomized controlled trials (RCTs). **Figure 2** also shows a systematic comparison of NAs, Peg-IFN, siRNA/ASO, therapeutic vaccines and PD-1/PD-L1 blockade in terms of their mechanisms, potential for functional cure, treatment duration, safety, cost and accessibility, and clinical positioning.

## 4 Predictive factors for functional cure

Achieving functional cure depends on a combination of viral, host, and immunological factors. Robust predictors are needed to optimize patient selection, define stopping rules, and guide the design of future trials. **Table 2** summarizes potential biomarkers associated with response to PD-1/PD-L1 blockade-based strategies.

**Table 2:** Potential predictors for HBsAg clearance in patients with CHB receiving PD-1/PD-L1 blockade therapy.

Predictor	Content	Study	Reference
Baseline HBsAg level	Low level, preferably less than 100 IU/mL	PD-1/PD-L1 blockade	(33, 34)
HBsAg kinetics	Substantial decline during treatment	PD-1/PD-L1 blockade	(30, 31, 35, 36)
ALT	ALT flare during treatment	PD-1/PD-L1 blockade	(31, 33, 34)
HBsAg-specific T cell responses	Enhanced HBsAg-specific T cell responses during treatment	PD-1/PD-L1 blockade	(31)
OAS1	Increased serum OAS1 level at baseline and during treatment	PD-1/PD-L1 blockade	(32)
Demographics	Younger age, female	Peg-IFN	(67)
Gene background	Gene polymorphisms in TP53BP2, IFN-γ	Peg-IFN	(65, 66)
Virological indicators	Genotype, HBsAb, quantitative hepatitis B core antibody, hepatitis B core-related antigen, large hepatitis B surface proteins, middle hepatitis B surface proteins	Peg-IFN	(67)
Biochemical indicators	Creatinine, hemoglobin, platelet	Peg-IFN	(67)
Immunological and other biomarkers	mMDSCs, CD4+ Treg, CD86+ pDC, CXCL9, microRNAs-3960, microRNAs-126-3p	Peg-IFN	(67)

ALT, alanine aminotransferase; CHB, chronic hepatitis B; CXCL9, C-X-C motif chemokine ligand 9; HBsAb, hepatitis B surface antibody; HBsAg, hepatitis B surface antigen; mMDSCs, Myeloid-derived suppressor cells; OAS1, 2′-5′-oligoadenylate synthetase 1; PD-1, programmed cell death protein 1; PD-L1, programmed death-ligand 1; pDC, plasmacytoid dendritic cell; Peg-IFN; pegylated interferon; TP53BP2, Tumor protein p53 binding protein 2.

### 4.1 Baseline HBsAg level

Low baseline HBsAg level is the most consistent enrichment marker for functional cure across Peg-IFNα therapies and emerging checkpoint regimens (22). A recent real-world Peg-IFNα study with the largest sample size found that HBsAg clearance rate correlates inversely with baseline levels: 6.9% for 1000–1500 IU/mL, 19.9% for 200–500 IU/mL, and rising to 53.6% for patients below 100 IU/mL (62).

PD-1/PD-L1 blockade appears to follow a similar antigen-threshold logic. In the study by Tak et al. (VTP-300 + nivolumab), HBsAg declines were most prominent in patients with baseline HBsAg <100 IU/mL (36). Similarly, the ASC22 trial by Qian et al. highlighted HBsAg 100 IU/mL as a critical threshold, with 42.9% (3/7) of patients in this sub-stratum achieving sustained HBsAg loss (34). Oncology study by Mon et al. corroborated this, reporting significantly higher cumulative HBsAg loss in the <100 IU/mL cohort (33).

Biologically, this supports a model where high antigen burden maintains a state of irreversible T-cell exhaustion (23, 41, 47). Only when the burden is low can checkpoint blockade effectively reinvigorate existing immunity, especially if it is primed by vaccination or interferon, enabling it to cross the threshold for durable clearance. However, studies by He et al. and Li et al. did not find a significant association between baseline HBsAg levels and response. This is likely due to very narrow range of baseline HBsAg levels in their cohorts (100–600 IU/mL in He’s and 2–84 IU/mL in Li’s study), where most patients had already crossed the major antigen bottleneck (31, 32).

### 4.2 HBsAg decline during treatment

As with interferon therapy, an early kinetic decline in HBsAg levels is a powerful predictor of a successful functional cure. In Peg-IFNα research, a rapid decline by week 12 is strongly associated with seroclearance, with an early decliner success rate of 54.8%, compared to 15.7% for those who did not decline early (62).

This pattern is mirrored in PD-1/PD-L1 trials. In He’s study, all three patients who achieved a functional cure with sintilimab exhibited a significant HBsAg reduction of > 2 log_10_ within the first 6 weeks (31). In the study by Gane et al., the single patient who achieved HBsAg loss demonstrated a 1 log_10_ IU/mL decrease by week 12 (30). Similarly, two HBsAg clearers in the study by Tak et al. showed an HBsAg decline exceeding 2 log_10_ by week 12 (36). In the sequential ropeg-IFN and nivolumab study by Qin et al., all three patients who achieved a functional cure showed a reduction of more than 1 log_10_ by the 12-week mark (35). Although Li et al. and Mon et al. did not explicitly analyze this correlation (32, 33), there is a general consensus that a functional cure is a continuous, dynamic process and that an absence of an early, robust decline can serve as a practical indicator of futility.

### 4.3 ALT flare

A recurring observation is that ALT flare, often viewed as adverse events in other contexts, frequently precede or accompany deep HBsAg decline and loss in the setting of immunotherapy (22, 63). This “good” immune flare likely represents the restoration of host cytolytic activity against infected hepatocytes (33).

According to He et al., ALT flare is an independent predictor of HBsAg clearance (multivariate analysis, β = 0.493, P = 0.006) (31). In the study by Qian et al., 57% of patients with >0.5 log_10_ HBsAg reduction and 67% of those with HBsAg loss experienced ALT flare (34). In oncology cohorts with CHB, 73% (13/17) of responders experienced immune-related hepatitis, with 61.5% of those achieving HBsAg loss (33). In these studies, ALT levels during flare typically peaked between 5–10 times the upper limit of normal (ULN), though some reached >10 times ULN. This flare was generally transient and self-limiting, typically resolving within 4 to 8 weeks without leading to hepatic decompensation.

### 4.4 Other biomarkers

Beyond routine clinical parameters, novel mechanistic markers are proving valuable. In the study by He et al., the fold-change in HBV-specific IFN-γ spots from week 0 to 12 (multivariate analysis, β = 0.409, *P* = 0.016) was significantly associated with HBsAg reduction. This provides direct evidence that the clinical signal is driven by T-cell reinvigoration (31). In the study by Li et al., levels of 2′-5′-oligoadenylate synthetase 1, a key ISG, were significantly higher at baseline and week 12 in HBsAg clearers compared to non-clearers (32). These findings suggest that a pre-existing or PD-1-potentiated ISG signature may determine the host’s capacity to achieve the “final push” towards functional cure (64).

Given that PD-1/PD-L1 blockade for CHB remains an emerging therapeutic strategy, clinical data are still limited, and more validated biomarkers or prediction models are not yet available. Future biomarker research may draw on previous studies of functional cure achieved with other therapies, particularly Peg-IFN. As shown in **Table 2**, predictors of Peg-IFN-related functional cure span multiple domains, including demographics, genetic background, virological indicators, biochemical parameters, and immunological or other biomarkers (65–67). While these factors cannot be directly applied to PD-1/PD-L1 blockade-based regimens, they could be useful for providing reference points in future prognostic studies of emerging cure-directed strategies.

## 5 Safety

The clinical deployment of PD-1/PD-L1 inhibitors for CHB requires a more favorable safety profile than is accepted in oncology. Recent trials suggest that, with optimized dosing, patient selection, and continuous NA protection, PD-1/PD-L1 blockade is both safe and manageable in carefully selected CHB populations (30–36). However, available data are still limited by small sample sizes and short follow-up, and continued safety evaluation is essential.

### 5.1 Adverse event profile

A notable consensus across recent CHB trials is that the incidence and severity of immune-related adverse events are significantly lower than those reported in oncology cohorts. This favorable safety profile is primarily driven by low-intensity strategies, characterized by reduced dosing (e.g., 0.3 mg/kg of nivolumab or a half-dose of sintilimab) and shorter treatment durations (typically ≤ 24 weeks). In the pilot study by Gane et al., a single low dose of nivolumab was well tolerated, with no serious adverse events (30). Similarly, in the 24-week sintilimab study by He et al., the vast majority of adverse events were Grade 1–2, with no treatment-related deaths or permanent discontinuations (31). The study by Tak et al. further confirmed the feasibility of combining PD-1 inhibitor with therapeutic vaccines without increasing the severe adverse events (36).

Non-hepatic immune-related adverse events, such as thyroid dysfunction and skin rashes, remain the most common side effects, in line with the wider experience of immune checkpoint inhibitors (ICIs) (30–36). However, these are generally manageable with standard protocols and rarely require systemic corticosteroids. Importantly, He et al. demonstrated that, even with a reduced dose, PD-1 receptor occupancy remained high (>90%) (31). This suggests that lower-intensity regimens can preserve the necessary biological activity for immune restoration while drastically reducing systemic toxicity.

Potential AEs of cure-directed strategies are summarized in **Figure 2**. Peg-IFN may cause flu-like symptoms, cytopenia, ALT flare, psychiatric effects, and autoimmune thyroid disease. siRNA/ASO-based therapies may be associated with injection-site reactions and ALT flare. Therapeutic vaccines are generally well tolerated but can cause local injection-site reactions and mild systemic symptoms. PD-1/PD-L1 blockade requires particular caution because immune activation may lead to immune-related adverse events, ALT flare, immune-mediated hepatitis, or HBV reactivation without adequate NA protection.

### 5.2 Immune-mediated liver injury and ALT flare

Checkpoint blockade in CHB carries a mechanistic risk of immune-mediated liver injury. Reinvigorated HBV-specific T cells can recognize infected hepatocytes through HLA-HBV peptide complexes and mediate hepatocyte killing via perforin/granzyme pathways and non-cytolytic IFN-gamma-dependent mechanisms. This can result in ALT flare, acute hepatitis, or rarely hepatic decompensation. The severity of liver injury may be influenced by antigen burden, pre-existing liver disease, and the intensity of T-cell reinvigoration (33, 48).

In several studies, transient ALT flare was associated with HBsAg decline or loss, suggesting that some flare may reflect beneficial target-mediated antiviral immunity (31, 33, 35, 36). Nonetheless, ALT flare should not be automatically regarded as favorable. Clinical management requires differentiation between controlled immune-mediated HBV flare, nonspecific drug-induced liver injury, autoimmune hepatitis, and HBV reactivation. Monitoring should include ALT, bilirubin, albumin, coagulation function, HBV DNA, HBsAg kinetics, and symptoms or signs of hepatic decompensation.

### 5.3 HBV reactivation

According to the AGA Clinical Practice Guidelines, PD-1/PD-L1 inhibitors are classified as a moderate-risk exposure (1%–10%) for HBV reactivation in HBsAg-positive individuals (28). Without antiviral prophylaxis, the risk is estimated at approximately 7%, whereas it is extremely rare in HBsAg-negative/anti-HBc-positive individuals (<0.1%). Concurrent NA prophylaxis reduces the risk of HBV reactivation to approximately 2% (29), a rate comparable to the background virological breakthrough observed during long-term NA monotherapy (68). Notably, none of the seven clinical studies reviewed here observed any cases of HBV reactivation. This favorable outcome is likely due to the strict requirement for viral suppression via long-term NA therapy prior to enrollment and the maintenance of NA treatment throughout the ICI exposure.

The biology of ICI-induced HBV reactivation remains complex (25, 41). While PD-1 blockade reinvigorates exhausted effector T cells to drive HBsAg loss, it may also disrupt hepatic immune homeostasis. PD-L1 negatively regulates regulatory T cells. Thus, its blockade could potentially lead to a disproportionate expansion of intrahepatic regulatory T cells. This could increase net immunosuppression and create a permissive environment for HBV replication, despite the simultaneous restoration of effector responses. This provides a biological explanation for HBVr in susceptible individuals.

Current evidence suggests that to navigate this paradox, a strict management protocol must be followed. The clinical exploration of ICIs for functional cure should be restricted to patients who are virally suppressed and have maintained uninterrupted NA therapy throughout their ICI exposure. This strategy successfully minimizes the risk of virological rebound while directing the “immune amplifier” effect of ICIs to be directed toward achieving a durable functional cure.

## 6 Unresolved questions and future directions

Although current evidence highlights the potential of PD-1/PD-L1 blockade for achieving functional cure, several key questions remain unresolved, including those concerning molecular targets, mechanisms of resistance, optimal dosing, treatment duration, combination strategies, and long-term durability.

### 6.1 PD-1 vs PD-L1

The choice of whether to target the PD-1 receptor on T cells or its ligand (PD-L1) on hepatocytes and antigen-presenting cells is still under investigation. Anti-PD-1 antibodies mechanistically reinvigorate exhausted CD8+ T cells directly, whereas anti-PD-L1 agents may more effectively disrupt inhibitory signals within the liver microenvironment. To date, most CHB trials have focused on PD-1 inhibitors, with only one study evaluating the subcutaneous PD-L1 inhibitor ASC22 (34). Head-to-head clinical comparisons are currently lacking. Real-world data from oncology cohorts with CHB suggested a slightly higher incidence of HBsAg loss with anti-PD-L1 than with anti-PD-1 (11.5% vs 6.6%), though this difference did not reach statistical significance (33). Overall, both approaches appear to be equally efficacy in driving HBsAg decline, though their safety profiles and tissue-specific penetration may differ (69).

### 6.2 Potential mechanisms of resistance or non-response

Some patients fail to achieve HBsAg loss even with optimized combination therapy. The PD-1/PD-L1 axis plays an important role in the development of immune exhaustion in CHB. However, other molecules also contribute to this exhaustion, such as TIM-3, LAG-3, CTLA-4, TIGIT, and 2B4 (37, 38, 46, 47). Blocking the PD-1/PD-L1 axis alone may not be sufficient to fully reinvigorate antiviral T-cell responses.

Persistent viral reservoirs also contribute to non-response: cccDNA remains a stable transcriptional template and integrated HBV DNA can contribute to sustained HBsAg expression independently of cccDNA. Importantly, HBsAg expression from integrated HBV DNA is influenced by the host genomic integration site and local chromatin environment. Current therapies are unable to specifically silence or eliminate this antigen source (7, 8). This may also explain why some patients fail to achieve HBsAg clearance despite potent viral suppression, antigen-lowering therapy, and checkpoint modulation.

### 6.3 Dosing optimization

A significant departure from oncology practice is the use of reduced doses in CHB patients. A pilot study by Gane et al. showed that low-dose nivolumab (0.1 or 0.3 mg/kg) achieved high peripheral receptor occupancy, which was sustained for up to 12 weeks in some patients (30). Similarly, He et al. confirmed that a half-dose of sintilimab maintained PD-1 receptor occupancy of over 90% (31), thus supporting the biological plausibility of low-intensity regimens. Although higher doses (3–10 mg/kg) were administrated to cancer patients, Qian et al. observed that a 1.0 mg/kg dose of the PD-L1 inhibitor ASC22 was more effective than a 2.5 mg/kg dose in reducing HBsAg (34). Given the small but real risk of triggering life-threatening autoimmune disorders in otherwise healthy CHB patients, the current trend favors reduced dosages that achieve immune restoration without exceeding the safety threshold.

### 6.4 Treatment duration and sequencing

The optimal treatment duration remains undefined. Short-course data show that even a single dose can trigger HBsAg decline (30, 36). In the study by He et al., significant HBsAg reduction occurred within the first 12 weeks, with a plateau observed thereafter despite continued treatment (31). In the study combining Peg-IFNα and PD-1 inhibitor, the median time to HBsAg loss was 12 weeks, ranging from 2 to 20.5 weeks (32). However, an analysis of the oncology cohort suggests that longer cycles may be necessary, particularly for patients with baseline HBsAg >100 IU/mL (33). These findings imply that patients with an ultra-low antigen burden could benefit from short-course rescue therapy, whereas those with a higher burden might require sustained immune pressure to overcome established immune tolerance.

### 6.5 Stability of functional cure

A critical question is how long HBsAg loss remains after the cessation of checkpoint blockade and combination therapy. While most patients who achieved HBsAg loss in the available studies maintained HBsAg negativity during follow-up, anti-HBs seroconversion rates were variable and follow-up was limited (30–36). Longer-term monitoring is required to determine whether immune-mediated control is stable and whether late relapse occurs.

### 6.6 Strategic combinations: the next frontier

The clearance rates achieved with single-agent PD-1/PD-L1 blockade remain modest. Current evidence increasingly supports the use of PD-1/PD-L1 blockade as part of a combination strategy rather than as a standalone intervention. The therapeutic logic is to integrate complementary mechanisms. NAs suppress HBV replication and reduce the risk of virological rebound. Peg-IFN provides direct antiviral and immunomodulatory effects. siRNA/ASO-based agents reduce HBsAg production and antigen burden. Therapeutic vaccines prime or enhance HBV-specific T-cell responses. PD-1/PD-L1 blockade functions as an immune amplifier that reinvigorates exhausted antiviral T cells. In a clinical study, Li et al. demonstrated that adding a PD-1 inhibitor to Peg-IFNα in IFN-treated patients increased the HBsAg clearance rate to 30.3% (32), suggesting that checkpoint inhibition may provide a “final push” toward seroclearance in patients who have already achieved substantial antigen reduction. The potential of this combination as a front-line cure-directed regimen, rather than only as a rescue strategy for IFN-refractory patients, is now under investigation (70). Preclinical evidence further supports this concept. An engineered liver-targeted anti-PD-L1-IFNα fusion protein combined with a therapeutic vaccine overcame vaccine resistance and promoted efficient HBsAg clearance in animal models (54). In parallel, antigen-reducing agents such as bepirovirsen may be another effective treatment option by lowering the HBsAg burden prior to immune-restorative intervention (71).

In line with this evolving framework, the ongoing clinical trials summarized in **Table 3** are exploring various combination strategies that incorporate PD-1/PD-L1 blockade. These include NA + PD-1/PD-L1 blockade, NA + Peg-IFN + checkpoint blockade, NA + siRNA + nivolumab, and triple regimens incorporating viral suppression, antigen reduction, and immune modulation. One phase 2 study, for example, is evaluating NA + JNJ-73763989, an siRNA targeting HBV mRNA, combined with one or three doses of nivolumab. This study will directly test whether antigen reduction can create a more permissive setting for checkpoint-mediated immune restoration. Other trials are evaluating combinations involving sintilimab, HLX10, cetrelimab, ASC22, nivolumab, or oral small-molecule PD-L1 inhibition. These efforts aim to optimize not only the combination partners, but also the route of administration, dosing intensity, treatment sequence and duration. Together, these studies will establish whether PD-1/PD-L1 blockade should be used as short-term rescue therapy, repeated immune-amplifying interventions or as part of a staged regimen following antigen reduction. They will also help to define which patients are most likely to benefit.

**Table 3:** Ongoing clinical studies evaluating PD-1/PD-L1 blockade for the treatment of CHB.

Trial number	Study design	PD-1/PD-L1 strategy	CHB Participants	Intervention	PD-1/PD-L1 dose
NCT05275023	Phase 2, open-label, RCT	NA + siRNA + PD-1/PD-L1 blockade	CHB	Arm 1: NA + JNJ-73763989 + nivolumab (1 dose) Arm 2: NA + JNJ-73763989 + nivolumab (3 dose) (JNJ-73763989: an siRNA targeting HBV mRNA)	0.3 mg/kg
NCT05771402	Phase 2, open-label, non-RCT	NA + Peg-IFN + PD-1/PD-L1 blockade	CHB	Arm 1: NA + anti-PD-1 antibody + Peg-IFNα Arm 2: NA + Peg-IFNα	Once/two or three weeks, dose lower than the dose used in cancer patients
NCT06457477	Open label, single arm	NA + Peg-IFN + PD-1/PD-L1 blockade	Virally suppressed with NA, Peg-IFNα treated	NAs + Peg-IFNα+ sintilimab	
NCT06357806	Open label, RCT	NA + PD-1/PD-L1 blockade NA + Peg-IFN + PD-1/PD-L1 blockade	Virally suppressed with NA	Arm 1: NA + Peg-IFNα Arm 2: NA + sintilimab Arm 3: NA + Peg-IFNα+ sintilimab	1.5 mg/kg sintilimab, once per 3 weeks for 24 weeks
NCT04133259	Phase 2, open label, single arm	NA + PD-1/PD-L1 blockade	HBV DNA < 2000 IU/ml with NA	NA + HLX10 (anti-PD-1 antibody)	1 mg/kg at 0, 4th, 8th week
NCT04638439	Phase 1, open label, single arm	NA + Peg-IFN + PD-1/PD-L1 blockade	HBV DNA < 2000 IU/mL (either under NA treatment or not)	entecavir + ropeginterferon alfa-2b + nivolumab	0.3 mg/kg nivolumab for 6 doses
NCT05242445	Phase 1, double blinded, RCT	NA + PD-1/PD-L1 blockade	NA treated for at least 6 mo	Arm 1: NA + cetrelimab (anti-PD-1 antibody) Arm 2: NA + placebo	Two doses
NCT05960240	Phase 1, single blinded, RCT	NA + PD-1/PD-L1 blockade	CHB	Arm 1: NA + AB-101 (an oral small molecule PD-L1 checkpoint inhibitor) Arm 2: NA + placebo	Administered orally
NCT07573943	Phase 4, open-label, RCT	NA + PD-1/PD-L1 blockade NA + Peg-IFN + PD-1/PD-L1 blockade	HBV DNA < 2000 IU/mL and HBsAg < 100 IU/mL	Arm 1: NA + ASC22 (anti-PD-L1 antibody) Arm 2: NA + Peg-IFNα + ASC22 Arm 3: NA + Peg-IFNα Arm 4: NA	Once two weeks, 1mg/kg, subcutaneous injection
NCT04225715	Phase 2, open-label, RCT	NA + siRNA + PD-1/PD-L1 blockade	Virally suppressed with NA	Arm 1: NA Arm 2: NA + CpAM + TLR7 Arm 3: NA + siRNA Arm 4: NA + siRNA + Peg-IFN Arm 5: NA + CpAM + siRNA Arm 6: NA + TLR7 + siRNA Arm 7: NA + siRNA + PD-L1 locked nucleic acid	Administered subcutaneously
NCT05343481	Phase 2, open-label, RCT	NA + vaccine + PD-1/PD-L1 blockade	HBV DNA < 1000 IU/mL with NA	Arm 1: NA + ChAdOx1-HBV, MVA-HBV and nivolumab Arm 2: ChAdOx1-HBV, MVA-HBV and nivolumab, MVA-HBV and nivolumab Arm 3: ChAdOx1-HBV, MVA-HBV, nivolumab, MVA-HBV	0.3 mg/kg nivolumab
NCT04891770	Phase 2, open-label, RCT	NA + siRNA + PD-1/PD-L1 blockade NA + siRNA + TLR8 + PD-1/PD-L1 blockade	CHB	Arm 1: TAF + VIR-2218 + selgantolimod + nivolumab Arm 2: TAF + selgantolimod + nivolumab (VIR-2218, an siRNA; selgantolimod, TLR8 agonist)	0.3 mg/kg intravenously Q4W for up to 24 weeks

CHB, chronic hepatitis B; HBV, hepatitis B virus; NA, nucleos(t)ide analogue; Peg-IFN, pegylated interferon; RCT, randomized controlled trial; siRNA, small interfering RNA; TLR, Toll-like receptor.

## 7 Conclusion

In conclusion, PD-1/PD-L1 blockade represents a promising but still investigational immunotherapeutic approach for CHB, particularly in patients who have achieved durable viral suppression with NAs and have a relatively low antigen burden. Although current antiviral therapies effectively suppress HBV replication, functional cure remains difficult to achieve because of persistent viral antigen production and long-standing immune dysfunction. PD-1/PD-L1 blockade has emerged as a strategy to partially overcome immune exhaustion, reinvigorate antiviral T-cell responses, and promote HBsAg decline or clearance. Current clinical studies evaluating nivolumab, sintilimab, ASC22, and related combination regimens have demonstrated encouraging but heterogeneous functional cure signals, with reported HBsAg loss rates ranging from approximately 2.5% to 30.3% across published studies, and higher responses generally observed in selected patients with low baseline HBsAg levels and in rational combination strategies (30–36). These findings suggest that PD-1/PD-L1 blockade may have the greatest therapeutic value as an adjunctive component of regimens that integrate viral suppression, antigen reduction, and immune priming, rather than as a standalone therapy. Nevertheless, major challenges remain, including optimal dosing, treatment duration, treatment sequencing, patient selection, biomarker development, and management of immune-related adverse events. Given the small sample sizes, heterogeneous study populations, and early-phase nature of the available evidence, large-scale multicenter RCTs are needed to further define the efficacy, safety, and clinical positioning of PD-1/PD-L1 blockade in CHB cure-directed therapy.
